# Harnessing Electrostatic Forces: A Review of Bees as Bioindicators for Particulate Matter Detection

**DOI:** 10.3390/insects16040373

**Published:** 2025-04-01

**Authors:** Simone Meacci, Lorenzo Corsi, Eleonora Santecchia, Sara Ruschioni

**Affiliations:** 1 Department of Agricultural, Food and Environmental Sciences, Polytechnic University of Marche, Via Brecce Bianche, 60131 Ancona, Italy; s.meacci@univpm.it (S.M.); l.corsi@univpm.it (L.C.); 2 Department of Industrial Engineering and Mathematical Sciences, Polytechnic University of Marche, Via Brecce Bianche, 60131 Ancona, Italy; e.santecchia@univpm.it

**Keywords:** bees, bioindicators, electrostatic forces, particulate matter adhesion, environmental monitoring, pollutants

## Abstract

Bees (Hymenoptera, Anthophila) are widely recognized for their essential ecological roles, including pollination and biodiversity maintenance. Recently, their ability to collect environmental particulate matter through electrostatic forces has been explored for biomonitoring purposes. This review integrates knowledge on electrostatic pollen adhesion with emerging insights into particulate matter adhesion to bees, emphasizing their potential as bioindicators. The mechanisms of electrostatic adhesion, influenced by factors such as the physicochemical properties of particulate matter and bee morphology, are discussed in detail. Additionally, the study evaluates the adhesion efficiency of pollutants, including heavy metals, microplastics, nanoplastics, pathogens, pesticides, radionuclides, and volatile organic compounds. This multidisciplinary approach underscores the role of bees in advancing environmental monitoring methodologies and offers innovative tools for assessing ecosystem health while addressing the drivers of bee decline.

## Simple Summary

This review examines the potential of bees, especially honey bees, as bioindicators for environmental particulate matter detection, utilizing their ability to collect particles through electrostatic forces. By drawing comparisons between pollen adhesion and particulate matter adhesion, the study investigates factors such as bee morphology and the physicochemical properties of particles. Key pollutants—including heavy metals, microplastics, nanoplastics, pathogens, pesticides, radionuclides, and volatile organic compounds—are assessed for their potential for electrostatic adhesion. The findings underscore the value of bees in monitoring environmental health and addressing biodiversity challenges, offering innovative methodologies for biomonitoring and ecosystem protection.

## 1. Introduction

Bees (Hymenoptera, Anthophila) are a globally distributed and diverse monophyletic taxon with over 20,000 described species [1,2]. Bees include several families, such as Andrenidae (e.g., mining bees), Apidae (e.g., bumble bees, carpenter bees, honey bees), Halictidae (e.g., sweat bees), Megachilidae (e.g., leafcutter bees, mason bees), among others [3]. These insects have been extensively studied due to their invaluable ecological roles; in particular, many bee species, especially wild bees [4,5], support wild plant communities and agriculture productivity through pollination service, thereby ensuring the maintenance of biodiversity and global food security [6,7].

In recent years, bees have also been investigated for their promising potential in biomonitoring [8]. Bees are found worldwide [1], and given their sensitivity to climatic [9] and environmental changes [10], coupled with the ease and low cost of sampling, as well as their well-known taxonomy and ecology, they are considered ideal bioindicators [8]. Through bee biomonitoring, it is possible to assess the qualitative status of the environment, including climate changes, habitat degradation and fragmentation, the degree of biodiversity, pollination efficiency, the presence of pollutants (e.g., heavy metals, microplastics and nanoplastics (MPs/NPs), pesticides, radionuclides, volatile organic compounds (VOCs)), and the spread of pathogens (e.g., bacteria, fungi, viruses) [11,12,13,14]. Wild bees are generally used less frequently as bioindicators than honey bees [11], particularly in biomonitoring related to climate changes [15], habitat degradation and fragmentation [11], and the degree of biodiversity [16], as they are highly sensitive to environmental changes due to their ecological specialization [11]. In contrast, honey bees are preferred as bioindicators because they can be economically bred, are abundant and widespread across various habitats, and are easier to recognize, sample, and manage in experimental settings [17,18,19,20]. Their use is primarily extended to biomonitoring of pollutants [14,21,22,23] and pathogens [14,24,25] dispersed in the environment (particulate matter). Indeed, honey bees travel long distances during foraging and can inadvertently collect particulate matter on their hairy bodies, bringing it back to the hive where it accumulates in honey, pollen, and wax, effectively acting as bioaccumulators [12,18,20,26,27,28]. However, using honey bees for biomonitoring may not fully capture the range of particulate matter exposure experienced by diverse wild bee populations [29,30]. Indeed, unlike wild bees, honey bees have distinct behavioral and ecological traits, and thus they may not interact with all environmental sources of particulate matter in the same way as wild bees [31]. For example, honey bees predominantly forage on flowers and do not frequently come into contact with soil or other non-floral surfaces where certain types of particulate matter may accumulate. Instead, wild bees display a wider variety of nesting habits, foraging strategies, and habitat use, often interacting with the soil [31,32]. Plus, honey bees possess distinct anatomical traits, such as body size, hair density, and leg morphology, that differ from those of many wild bee species, potentially affecting their ability to trap and transport certain types of particulate matter. Additionally, it is not possible to introduce honey bees into areas where they are absent without considering their potential impact on biodiversity [33]. Therefore, in certain environmental contexts, the use of wild bees may be preferred. For instance, bumblebees have been used to detect heavy metals [34], and Megachilidae have been employed to detect pesticides [35].

The ability of bees to collect particulate matter on their bodies is, at least in part, the result of its adhesion due to electrostatic forces [36,37], similarly to the process of pollen adhesion [13]. Electrostatic forces generally promote pollen adhesion to bees when they visit flowers, facilitating pollination and playing a crucial role in bee ecology [38,39,40,41,42,43,44]. In this context, an approach to bee biomonitoring based on electrostatic forces could be an innovative and powerful tool when applied to particulate matter detection. Indeed, a better understanding of how electrostatic forces are involved in bee ecology, and how these forces are influenced by the physicochemical properties of particulate matter and bee morphology, could be valuable for designing more effective experimental protocols and identifying new types of particulate matter detectable via bee biomonitoring. This would enable the collection of more meaningful data on environmental conditions and the factors driving bee decline. To this end, this review will first discuss electrostatic pollen adhesion to bees in detail, as a valid model for understanding electrostatic particulate matter adhesion to bees. Second, this information will be used to describe how and which types of particulate matter may electrostatically adhere to bees, based on their physicochemical properties. However, before proceeding, it is important to clarify that, at least in bumblebees and honey bees, electrostatic forces are involved not only in pollen adhesion but also in other aspects of bee ecology, such as the communication through electroreception (i.e., the perception of external electric fields, a phenomenon common to many animals [45,46] and the electroreception of flowers [40].

## 2. Search Strategy and Selection Criteria

To ensure a comprehensive and relevant selection of studies, we employed a systematic search strategy across multiple key databases, including Google Scholar, PubMed, ScienceDirect, Scopus, and Web of Science. The search was conducted using specific keywords relevant to the topic, such as “bees particulate matter detection”, “bees biomonitoring”, “bees electrostatic force”, and “bees electrostatic adhesion”. Studies were selected based on predefined inclusion criteria, prioritizing research focused on the biomonitoring of particulate matter using bees and electrostatic force. The selected studies were then ranked according to their relevance to the research question, the rigor of their methodology, and their contribution to the existing body of knowledge. While recognizing the value of other review methodologies, such as meta-analysis or systematic reviews, we opted for a narrative review approach due to the heterogeneity of the included studies. Our aim was to provide a comprehensive overview of the current state of research rather than a statistical synthesis.

## 3. Electrostatic Pollen Adhesion to Bees

**Figure 1:**
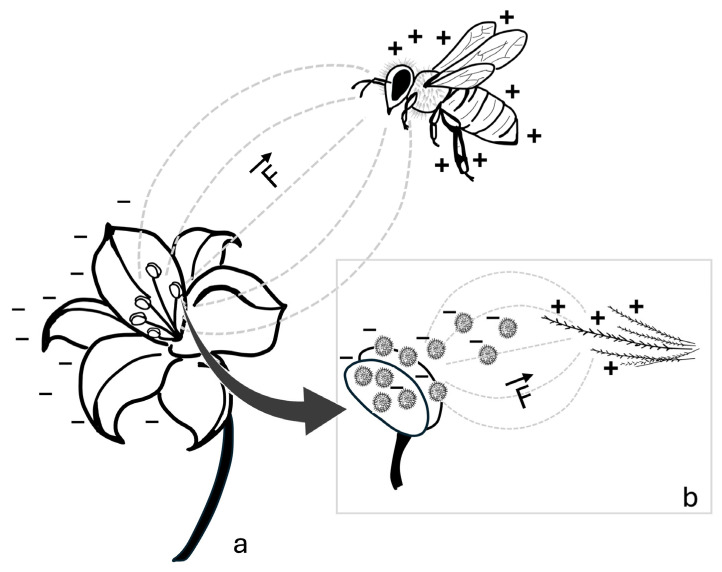
Schematic representation of the electrostatic interaction between a bee and a flower. (**a**) The potential difference between the positively charged bee and the negatively charged flower generates an interaction of electric fields (---), resulting in an electrostatic force (F). (**b**) Enlarged detail showing negatively charged pollen grains detaching from the negatively charged anthers and being electrostatically attracted to the positively charged branched hairs of the bee. The direction and orientation of the electrostatic force are indicated by the vector (→). Symbols: +, positive charge; −, negative charge.

Like other flying insects, bees acquire a positive electric potential during flight [40]. Although the underlying mechanisms of this phenomenon are not yet fully understood, it is partially attributed to the triboelectric effect [41], which refers to the transfer of electric charge between materials that come into contact or slide against each other [47]. Bees gain a positive electric potential by losing electrons through aerodynamic friction during flight, as the atmosphere is positively charged under clear weather conditions [41,48]. In contrast, negative charges continuously migrate from the atmosphere to the ground, causing earthed objects, such as flowers, to acquire a negative electric potential [41,49]. As illustrated in Figure 1, when a bee approaches a flower, the difference in electric potential between the bee and the flower generates two interacting electric fields [39,40]. This interaction is known as the electrostatic force or Coulomb force, which arises due to the behavior of electric charges: when one charge is positive and the other is negative, they attract each other, whereas like charges (either both negative or both positive) result in a repulsive force between the interacting bodies [50]. The electrostatic force (*F*) is governed by the Coulomb law, expressed as:

$$F=k\frac{\left|q_{1}\right|\left|q_{2}\right|}{d^{2}}$$

In this equation, *k* represents the proportionality constant (*k* = 8.987 × 10^9^ N × m^2^/C^2^), which determines the magnitude of the force in newtons (N); *q1* and *q2* denote the interacting bodies approximated as point charges in coulombs (C); *d*^2^ represents the square of the linear distance between the two bodies in meters (m) [43,46,50,51]. Thus, the intensity of electrostatic force depends on the magnitude of the charges and reaches its maximum at the shortest distance between the two bodies. Consequently, at very short distances, the positive charges on the bee induce a strong negative polarization in the flower in the direction of the bee. As a result, pollen, which is also negatively charged, is attracted to the positively charged bee until it adheres electrostatically to the insect [38,39,40,41,42,43,44].

More specifically, electrostatic pollen adhesion is influenced by weather conditions [49], gravity [52], and, most notably, two key factors: the chemical composition of pollen [53] and the morphology of the interacting bodies (i.e., the flower, the pollen, and the bee) [41,42,54]. Understanding these factors is essential for enhancing current knowledge on bee-based biomonitoring on particulate matter, as they may also affect electrostatic particulate matter adhesion to bees. Therefore, the following sections will examine their impact on electrostatic pollen adhesion in detail.

### 3.1. Pollen Chemical Composition

At the chemical level, the primary contributor to electrostatic pollen adhesion appears to be the chemical composition of sporopollenin, which is the main constituent of exine (i.e., the outer layer of pollen grains [55]). Experimental studies on synthetic materials derived from sporopollenin have demonstrated that some functional groups contained in this substance, such as amino (-NH_2_) and carboxyl (-COOH) groups, can ionize, and acquire an electric charge [56]. Moreover, environmental pH plays a crucial role in this ionization process [56,57]. Additionally, charged sporopollenin has been observed to electrostatically adsorb various substances, including copper atoms [57] and proteins [58]. Similarly to pollen, different types of particulate matter may exhibit distinct electrostatic behaviors, due to their chemical composition.

### 3.2. Morphology of the Interacting Bodies

**Figure 2:**
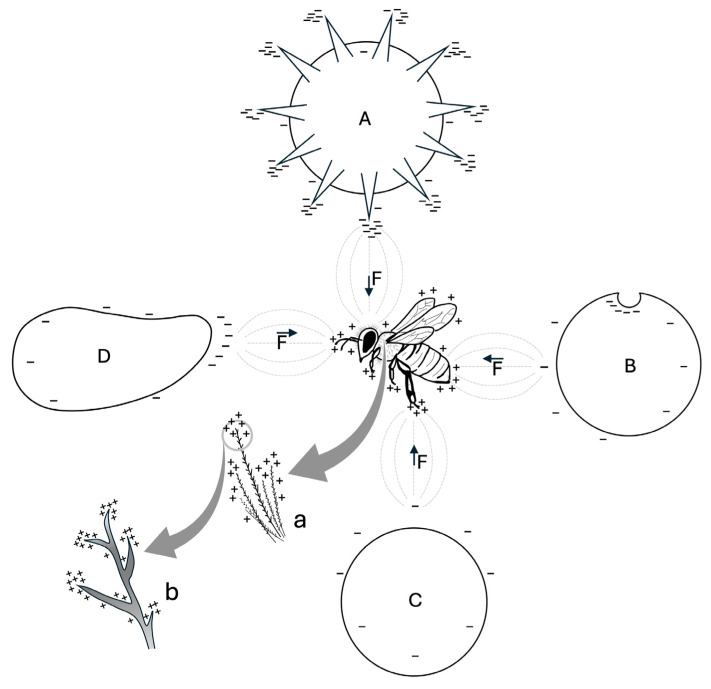
Effect of morphology on the distribution of positive (+) and negative (−) surface charge density on a bee and particles with different shapes (A, B, C, D). Due to point discharge, the radius of surface curvature determines the accumulation of electric charges on the objects shown. On the bee, positive charges accumulate primarily on the legs, antennae, and branched hairs. On the particles, negative charges are more concentrated on spines, concavities, and other small structures: echinate pollen grain (A); concave particle (B); rounded particle with lower surface charge density (C); elongated particle (D). The interaction between the electric fields (---) of the bee and each particle generates an electrostatic force (F), with direction and orientation indicated by the vector (→). (**a**) magnification of hairs; (**b**) magnification in which it is shown the higher concentration of positive charges on the tip of the hair branches.

The morphology of the bodies interacting within the “flower-pollen-bee system” influences the distribution of the electric fields around them, and, consequently, the strength of electrostatic force [41,54], as illustrated in Figure 2. The surface charge and, consequently, the surface charge density are inversely correlated with the radius of surface curvature. As a result, a smaller radius (in the most extreme case, a sharp point) exhibits a higher surface charge density [59]. This phenomenon is known as the “point discharge” or “point effect”.

As a result, on the flower, the charge density increases along contours and sharp components (e.g., anthers, edges of petals, stigma) as well as on small components (e.g., trichomes) [41,54]. This mechanism favors the transfer of negatively charged pollen from the anthers to the bee, and from the bee to the stigmas, when the insect visits other flowers [49]. This process may also be crucial for electrostatic particulate matter adhesion.

Similarly, as a result of point discharge, the sculpture of pollen grains—the protuberances, concavities, and grooves on their exine—may enhance the charge density on their surface. For example, it has been proposed that a protruding pollen morphology, such as the echinate sculpture (i.e., covered with external spines), may promote electrostatic pollen adhesion to the stigma and to pollinators [52,60] (see Figure 2). Additionally, these structures increase the contact area with the substrate, thereby amplifying van der Waals forces [61,62]. Van der Waals forces are defined as interactions between atoms and molecules, separated by any medium (including air or vacuum), based on their mutual distance. The electrostatic interactions between the shifting electron clouds that comprise molecules and other materials give rise to these nonbonded forces, which have a quantum mechanical origin [63]. These dispersive forces—comprising Keesom, Debye, and London contributions—are most active at relatively short distances compared to charge–charge interactions (approximately 10 nm at the nanoscale or even farther in materials at the mesoscopic level) [63]. Van der Waals forces, together with the sculpture of exine and viscous substances (e.g., pollenkitt), are involved in the mechanism of pollen retention/detachment in/from anthers [62,64,65], and promote pollen adhesion to experimental surfaces [66] and to the stigma [67]. Therefore, van der Waals forces may also work in synergy with electrostatic force in pollen adhesion to the body of bees, as both forces are significant for particles with diameter <100 μm [68]. However, while electrostatic force dominates for particles >50 µm, van der Waals forces influence smaller particles [61], and these forces are amplified with particle size reduction [69]. Thus, the influence of electrostatic force and van der Waals forces on pollen may change with particle size, as each plant species produces pollen grains of varying sizes, ranging from less than 10 µm to over 100 µm [70]. Moreover, the weight of pollen grains must be considered, as lighter pollen grains are more likely to overcome gravity, and be affected by electrostatic force [52]. These findings align with the assumptions of Zeghloul et al. (2017) [71]: the smaller and lighter the particles, the higher the ratio of electrostatic force to gravitational force, making their electrostatic separation easier. Consequently, as with pollen, both the morphology and the size of particles are crucial for electrostatic particulate matter adhesion.

Lastly, bee morphology plays a significant role in electrostatic pollen adhesion [38,42,72,73,74]. Bees are covered by millions of branched hairs, which are essential for mechanical pollen adhesion and transport [75,76,77,78], but are also electrostatically charged [79,80]. These hairs may enhance electrostatic pollen adhesion through point discharge: since hair branches can be approximated to thin points, they may exhibit a high charge density on their surface. Additionally, the numerosity of these hairs can amplify this phenomenon, particularly in bee species with wide hairy areas (see Figure 2). Furthermore, similar to the cuticle of other insects [81], the bee cuticle may possess electrostatic characteristics, potentially contributing to both electrostatic pollen and particulate matter adhesion.

Having outlined the process of electrostatic pollen adhesion and the key factors involved—the chemical composition of pollen and the morphology of the interacting bodies—it is now possible to apply this information to electrostatic particulate matter adhesion to bees. In the following paragraph, particulate matter will be analyzed in terms of physicochemical properties that may promote its electrostatic adhesion to bees and its detection via bee biomonitoring.

## 4. Analysis of the Physicochemical Properties Involved in Electrostatic Particulate Matter Adhesion to Bees

In this paragraph, the three hypothetical modalities of electrostatic particulate adhesion to bees—direct, indirect, and combined—will be briefly described. Next, the efficiency of electrostatic adhesion of certain types of particulate matter to bees, including heavy metals, MPs/NPs, pathogens, pesticides, radionuclides, and VOCs, will be discussed with reference to the most recent research on their physicochemical properties. As it is not feasible to cover all types of particulate matter, only select examples from each category of particulate matter will be presented.

### 4.1. Modalities of Electrostatic Particulate Matter Adhesion to Bees

Following the assumptions of Perugini et al. (2011) [82], it can be hypothesized that various types of particulate matter may adhere electrostatically to bees through at least three modes: the first is direct electrostatic adhesion, in which particulate matter adheres to the bee’s body due to its electrostatic charge; the second is indirect electrostatic adhesion, wherein particulate matter binds to an intermediary substance—such as pollen [83] or other materials—that may adhere to the bee, or that has already adhered to the bee and can further attract particulate matter [84]; the third is combined electrostatic adhesion, which results from the synergistic effect of the bee’s morphological electrostatic properties and the electrostatic properties of intermediary substances accumulated on its body. These three mechanisms may operate simultaneously, with their relative intensity varying depending on the physicochemical properties of each type of particulate matter, as discussed below.

### 4.2. Heavy Metals

Heavy metals primarily occur in dispersed ionic form [85] or as constituents of other pollutants [86,87]. They are continuously released from both natural sources and anthropogenic activities—including agriculture, industries, mining, and transportation—becoming hazardous pollutants due to their non-biodegradable nature and their ability to enter food chains, where they exert toxic effects on organisms [88,89,90,91]. While some heavy metals are essential for biological functions, they become toxic in excess, whereas others are harmful even at low concentrations [88,91].

Due to these concerns, environmental pollution by heavy metals has been extensively studied through bee biomonitoring [82,92,93,94,95,96,97,98,99,100,101,102,103,104,105,106]. Indeed, atmospheric heavy metals can adhere directly to bees and/or indirectly reach them via contaminated pollen [82]. In particular, direct adhesion may be partially driven by electrostatic force, as heavy metals are often present as ions, similar to what has been observed in studies on microorganisms. Research suggests that electrostatic interactions contribute to microbial cell walls via functional groups such as carboxyl, amino, hydroxyl, phosphate, thiol [107,108,109]. Similarly, indirect and combined adhesion via pollen attached to bees may be facilitated by electrostatic force. Supporting this hypothesis, sporopollenin has been observed to electrostatically adsorb copper atoms [57]. Finally, heavy metals may reach bees through electrostatic adhesion of pollutants that contain them, such as MPs/NPs [86] and pesticides [87].

### 4.3. Microplastics and Nanoplastics

MPs/NPs originate either from the degradation of various plastic materials or as microcomponents intentionally produced for industrial and cosmetic applications [110,111]. Owing to their diverse origins, each type of MPs/NPs exhibits unique chemical composition, shape (e.g., fibers, films, fragments), size, and weight, resulting in heterogeneous physicochemical properties [110,112,113,114]. These properties contribute to their exceptionally long degradation time [115], rendering MPs/NPs nearly ubiquitous in the environment. As a consequence, they pose a serious threat to organisms by infiltrating biological tissues and inducing adverse effects such as toxicity and metabolic disturbances, which remain incompletely understood [116,117,118]. Additionally, MPs/NPs can be transferred through food chains [119,120,121], release toxic additives and heavy metals into the environment [86,112,113], and serve as substrate for biofilms of pathogenic microorganisms [122,123].

Despite the severity of MPs/NPs pollution, research on their impact in the terrestrial environment remains limited, whereas their presence and effects have been extensively studied in aquatic ecosystems [119,124,125,126]. Consequently, studies investigating bees themselves as bioindicators of MPs/NPs are still scarce [13,127,128]. However, bees have been observed to accumulate MPs/NPs on their body [13], likely due to electrostatic force, given that MPs/NPs have been detected in the atmosphere [129], on flowers [130], and, hypothetically, on pollen [131,132]. Pollen may, therefore, act as an intermediate in indirect and combined electrostatic adhesion of MPs/NPs to bees. While no direct evidence currently confirms electrostatic MPs/NPs adhesion to bees, this hypothesis is supported by studies demonstrating electrostatic adhesion of MPs/NPs to algae and plants [133,134,135,136]. Furthermore, Wang et al. (2022) [137] described MPs/NPs adhesion to soil as a result of both electrostatic and van der Waals forces, suggesting that these forces may also act in synergy to facilitate the electrostatic adhesion of pollen and particulate matter to bees. Given the heterogeneity of MPs/NPs in terms of chemical composition, size, and shape, the relative contributions of electrostatic force and their interaction with van der Waals forces in MPs/NPs adhesion to bees may vary depending on the specific characteristics of the particles.

For instance, the chemical composition of MPs/NPs may influence their electrostatic adhesion to bees, depending on the type of functional groups present on their surface and/or the presence of additives and their respective functional groups. Indeed, some of these functional groups may ionize and acquire a charge similar to those found in sporopollenin [56]. Furthermore, the chemical composition of MPs/NPs can determine their charge polarity—either positive or negative—as suggested by the study of Park et al. (2008) [138] on various plastic materials. Consequently, this charge polarity may influence the electrostatic adhesion of MPs/NPs to bees. As indicated by Park et al. (2008) [138], plastic materials exhibit specific charge tendency in a triboelectric series (i.e., a ranking of materials based on their propensity to gain or lose electrons [139]). The ranking is as follows, from negative to positive: Polychloroethylene with a high polymerization degree (HPVC)—Suspension Polyvinyl Chloride Resin (SPVC)—Copolymer Polypropylene (COPP)—Homopolypropylene (HOMOPP)—Low Density Polyethylene (LDPE)—High Density Polyethylene (HDPE)—Polyethylene Terephthalate (PET)—Rubber—High Impact Polystyrene (HIPS)—Calibre—Acrylonitrile Butadiene Styrene (ABS)—General Purpose Polystyrene (GPPS)—Poly (Methyl Methacrylate) (PMMA). Since bees carry a positive charge, it is plausible that they preferentially attract MPs/NPs originating from the most negatively charged plastic materials in this triboelectric series.

Regarding the shape of MPs/NPs, it may determine the strength of electrostatic force, considering that pollen adhesion to bees is influenced by its morphology (see Figure 2). Therefore, it is plausible that the sharpest and/or thinnest MPs/NPs, such as fibers, are the most involved in electrostatic adhesion due to the point discharge effect (see Figure 2). This hypothesis may partially explain why fibers were the most abundant MPs/NPs shape in the study by Cortés-Corrales et al. (2024) [127], although Edo et al. (2021) [13] primarily found fragments. However, these contrasting results could be attributed to differences in sampling sites [127] or to the high irregularity and sharpness of the collected fragments, which may enhance point discharge, and consequently, electrostatic adhesion (see Figure 2). Indeed, most MPs/NPs dispersed in the environment exhibit an irregular shape, as they originate from the degradation of plastic materials [140]. Additionally, certain MPs/NPs, such as glitters, feature highly irregular and angular geometries (e.g., snowflakes, squares, stars, triangles) [141], which may further enhance point discharge and, therefore, electrostatic adhesion.

Lastly, the size of MPs/NPs may influence their susceptibility to electrostatic force and the interplay between electrostatic and van der Waals forces, similarly to what occurs with pollen grains. Additionally, the weight of MPs/NPs may affect their resistance to electrostatic adhesion. As observed for certain pollen grains, smaller and lighter MPs/NPs are more likely to adhere electrostatically to bees. Indeed, Zeghloul et al. (2017) [71] stated that the size of MPs/NPs influences their electrostatic separation.

### 4.4. Pathogens

Pathogens—including fungi, bacteria, and viruses—are of significant interest to humans due to their direct impact on human health [142] and their indirect effects on society [143]. Additionally, many pathogens affect domestic animals and cultivated plants, leading to individual losses, reduced product quality, significant damage to agricultural production, and the transmission of zoonotic diseases [144,145]. The threat of pathogen spread is even extended to bees. Beyond the honey bee mite *Varroa destructor* [146], several pathogens affect these insects, causing behavioral and metabolic changes and, in severe cases, death [147,148,149,150,151].

Consequently, in recent years, the emergence and spread of pathogens have been widely studied due to their high impact on both humans and other organisms, including bees. The recent global SARS-CoV-2 pandemic has underscored the urgency of constantly monitoring the presence of airborne pathogens in the environment. However, unlike foodborne and waterborne pathogens, research of airborne pathogen detection remains limited [152]. A promising approach for detecting airborne pathogens and studying their emergence trends is bee biomonitoring. Indeed, bees have been successfully employed to study the environmental presence of airborne entomo-, human-, and phytopathogens, including bacteria [153,154,155], fungi and oomycetes [156,157], and viruses [158,159,160]. Pathogen biomonitoring through bees is feasible because these insects can passively transport various microorganisms on their bodies, contributing to plant infections as well [14,161,162,163]. It is likely that pathogens adhere directly to bees at least partially due to electrostatic force. Indeed, electrostatic force—along with van der Waals forces and other mechanisms—has been shown to play a role in the adhesion of bacteria [164,165,166,167,168,169], fungi [170,171,172,173], and viruses [174,175,176,177,178] to surfaces. Furthermore, since bacteria [179], fungi [162], and viruses [162] can be transmitted via pollen [162,180], pollen attached to bees may electrostatically adsorb pathogens on its surface, facilitating their secondary transport to bees. Therefore, for pathogens, indirect and combined electrostatic adhesion via pollen may be particularly relevant, although further research is needed to confirm this hypothesis.

Regarding the physicochemical properties that may facilitate electrostatic pathogen adhesion to bees, chemical composition could be relevant, at least for viruses. In fact, the electrostatic adhesion of viruses to pollen grains may involve the proteins forming the viral capsid—an external protein shell that protects the genome [181]—since, as mentioned earlier, sporopollenin has been shown to electrostatically adsorb proteins [58]. Moreover, morphology may also play a crucial role, as certain pathogen structures could enhance point discharge and, consequently, electrostatic pathogen adhesion to bees (see Figure 2). For instance, the external ornamentations of some fungal spores, suggested to facilitate adhesion to arthropod vectors [182,183], could be among these candidate structures. Another example is the spike proteins forming small protrusions on the capsid of SARS-CoV-2 and other coronaviruses [184]. These glycoproteins mediate a complex process of host cell recognition, binding, membrane fusion, and viral entry [184]. In this process, electrostatic force arising from the charges accumulated on spike proteins, along with van der Waals forces, is also involved [175,176,177,178]. Therefore, the spherical and crown-like SARS-CoV-2 virions (i.e., individual viral particles [185]), which may be compared to pollen grains with echinate sculptures, could electrostatically adhere to bees due to point discharge effects resulting from their morphology (see Figure 2). This hypothesis may partially explain the successful detection of SARS-CoV-2 through bee biomonitoring, as reported by Cilia et al. (2022) [159].

### 4.5. Pesticides

Pesticides pose a significant threat to the environment and living organisms due to their chemical composition and their extensive, unselective, excessive, and incorrect use [186]. They are often toxic to a wide range of non-target organisms, causing poisoning and metabolic disturbances, and they can be transferred through food chains [186]. In particular, inorganic pesticides are especially hazardous, as they frequently contain heavy metals that can be released into the environment [187]. Even pollinators, such as bees, are not exempt from the harmful effect of pesticides, both directly—since many pesticides are toxic to these insects—and indirectly, as herbicides can lead to the destruction of plant communities [186,188].

Due to their high toxicity, which endangers pollinator diversity and their crucial ecological role, pesticides have been extensively monitored through bee biomonitoring studies [17,29,30,99,189,190,191,192]. In particular, the direct adhesion of pesticides to bees may, at least for certain compound classes, occur partially through electrostatic force. For instance, in the 1970s, the insecticide Penncap M, composed of capsules the size of pollen grains, electrostatically adhered to bees, until its subsequent ban [193]. Furthermore, in an effort to reduce dosage requirements, many pesticides have been optimized through air-assisted electrostatic spraying techniques, which enhance their adhesion to target surfaces [193,194,195,196,197]. However, this process may also result in the unintended electrostatic adhesion of electrified pesticide droplets to bees and other non-target organisms [193]. This effect may be further intensified by point discharge (see Figure 2), as droplets can preferentially adhere to the narrowest plant surfaces, such as floral structures visited by bees [193]. Moreover, chemical composition may play a relevant role in the indirect and combined electrostatic adhesion of pesticides to bees. Certain pesticide chemical components, such as heavy metals [57], may be electrostatically adsorbed by pollen grains, which specific functional groups may ionize and generate a charge, similarly to the functional groups of sporopollenin [56]. Finally, many pesticides, such as tetraconazole, fipronil, and deltamethrin, have been found to adsorb onto atmospheric particles [198], potentially leading to their electrostatic adhesion to bees via these substances.

### 4.6. Radionuclides

Having an excess number of neutrons or protons, some atoms, called radionuclides, randomly decay into more stable atoms by releasing ionizing radiation [199,200]. This phenomenon is referred to as radioactivity or radioactive decay [199,201]. While radionuclides are a natural occurrence, various anthropogenic sources—including mining, nuclear power plants, and the historical use of nuclear weapons—now contribute to radionuclide pollution [202,203]. This type of pollution is particularly hazardous for organisms, as ionizing radiation acts as a powerful mutagen, inducing heritable mutations in the genome [204,205], which can lead to cancers and teratologies [206,207]. Additionally, some radionuclides can enter organisms and be transferred along food chains [208,209].

Due to the significant risks associated with radionuclides, advancing detection methods for these substances in the environment is crucial. Biomonitoring using bees has emerged as a promising approach, as bees actively collect environmental particles over large areas and can serve as effective indicators of contamination. Several studies have demonstrated the potential of this method for radionuclide detection [100,210,211,212,213,214,215,216,217]. Radionuclide bee biomonitoring may be facilitated by electrostatic adhesion of radionuclides to bees or, more likely, of particles containing them. Indeed, radionuclides are often found in many atmospheric particles [218], and their radioactivity is known to induce a charge on these particles, thereby enhancing electrostatic particle interactions [219,220,221,222,223,224,225,226]. Radionuclides may also reach bees via pollen through electrostatic adhesion, and their radioactivity may increase the charge of contaminated pollen grains, facilitating their electrostatic adhesion to bees. This hypothesis is supported by the findings of Tschiersch et al. (1999) [227], who observed high concentrations of ^137^Cs on pine pollen grains after the Chernobyl nuclear accident. Therefore, for radionuclides, indirect and combined electrostatic adhesion to bees may be particularly relevant.

### 4.7. Volatile Organic Compounds

VOCs are characterized by low boiling points, which allows many of them to easily evaporate into the atmosphere [228,229]. This class of inert compounds includes acids, alcohols, aliphatics, aldehydes, ethers, hydrocarbons, and ketones [228]. While these substances can evaporate from natural sources, anthropogenic sources—such as gas and petroleum extraction, industrial and residential coal burnings, means of transport, paints, printers [228,230,231], and silage [232]—release a relevant amount of VOCs [228], which can be toxic to organisms [233,234]. For instance, in humans, their toxicity is associated with metabolic [231,235] and respiratory illnesses [231,236,237], reproductive defects [231], and even cancer [229,231,234,238,239]. Additionally, VOCs emissions have an indirect effect on climate change, as they contribute to the reduction of stratospheric ozone—an important greenhouse gas—and participate in photochemical reactions that produce tropospheric ozone [229,240,241].

Due to their substantial impact on the environment and organisms, VOCs have been extensively biomonitored, and several studies have utilized bees as bioindicators for these substances [105,242,243,244,245,246,247,248,249]. For VOCs, indirect and combined electrostatic adhesion to bees may be the most significant mode of adhesion, considering that some VOCs, such as aromatic and aliphatic hydrocarbons, are associated with atmospheric particles [250,251], which may transport them to bees. Chemical composition may also be a relevant factor for their electrostatic adhesion to bees. Specifically, this phenomenon may occur if certain functional groups of these substances, such as amino and carboxyl groups, ionize, thereby generating a charge. This mechanism may be similar to the ionization of functional groups observed in sporopollenin [56] and hypothesized for MPs/NPs and pesticides.

## 5. Conclusions

This review highlights the crucial role of bees as environmental bioindicators, with a particular emphasis on their ability to collect particulate matter through electrostatic forces. By analyzing the mechanisms of electrostatic adhesion and its implications, the review illustrates how various types of particulate matter—including heavy metals, MPs/NPs, pathogens, pesticides, radionuclides, and VOCs—can adhere to bees and, consequently, be used for monitoring environmental quality.

One of the key findings is that electrostatic adhesion is influenced by multiple factors, including the electric charge acquired by bees during flight and the specific physicochemical properties of each type of particulate matter. The significance of these properties may vary for different types of particulate matter. For instance, in the case of heavy metals, shape, size, and weight may be less critical than their ionic properties. Hypothetically, each type of particulate matter may follow a distinct mode of electrostatic adhesion: direct, indirect, and combined electrostatic adhesion may be more relevant for heavy metals, MPs/NPs, pathogens, and pesticides, whereas indirect and combined electrostatic adhesion may be more relevant for radionuclides and VOCs.

However, the true relevance of physicochemical properties in particulate matter electrostatic adhesion remains unknown and should be investigated through experimental studies. Meaningful experiments should involve particles from the same category with varying properties as well as particles from different categories, employing a multidisciplinary approach combining Ecology, Physics, and Chemistry. This will enable the testing of the hypotheses proposed in this review, which may serve as a valuable starting point for understanding particulate matter electrostatic adhesion to bees and its potential applications.

This knowledge may open new perspectives for biomonitoring by leveraging the natural interactions between bees and airborne pollutants, potentially providing a more refined and sensitive approach compared to traditional monitoring methods. The proposed methodology may be essential for refining detection methods, identifying new environmental contaminants, and improving biomonitoring accuracy by enhancing pollutant detection, especially for substances difficult to monitor conventionally. Moreover, it may provide a better understanding of pollution sources and their distribution, improving risk assessments for environmental and human health. However, given the wide ecological, behavioral, and morphological differences among bee species, their effectiveness in biomonitoring through electrostatic force must be investigated to identify the most suitable species for the detection of each type of particulate matter. In this way, alongside the practical and cost-effective use of honey bees, particulate matter biomonitoring could be extended through the use of various wild bee species, each potentially capable of collecting different types of particulate matter. Ideally, using a combination of different bee species as particulate matter bioindicators may offer a more comprehensive understanding of the pollution levels in a given area.

Furthermore, these findings carry important implications for biodiversity conservation. This approach could be particularly valuable for bee conservation, as many types of particulate matter can negatively impact these pollinators. For example, exposure to heavy metals and pesticides may disrupt bee behavior, reduce reproductive success, and undermine their ecological role in pollination. However, the severity of these effects can vary among bee species, as each species—due to its unique ecological, behavioral, and morphological traits—may exhibit different levels of resistance and face varying degrees of exposure to particulate matter. Pollutants not only affect pollinators directly but can have cascading effects across ecosystems. The decline of honey bee and wild bee populations due to pollutant exposure could reduce floral diversity and decrease agricultural yields dependent on pollination. Additionally, radionuclides and VOCs may disrupt plant-pollinator attraction dynamics, undermining the effectiveness of pollination as a critical ecosystem service. Plus, studies suggest that MPs/NPs accumulation in terrestrial ecosystems could degrade soil quality, affecting food sources for pollinators and disrupting trophic networks. In this context, by identifying areas with high pollution exposure, this approach could help locate regions where wild pollinators are most at risk, guiding targeted conservation efforts. Given the threats posed by pollutants like pesticides and heavy metals to pollinators, incorporating electrostatic biomonitoring into environmental assessments can contribute to more effective mitigation measures.

In conclusion, the use of bees as bioindicators represents a valuable opportunity for both pollution monitoring and biodiversity conservation. Bees can not only provide crucial data on the presence and spread of environmental contaminants, but also help identify high-risk areas where biodiversity is particularly threatened. Therefore, integrating electrostatic principles into biomonitoring presents a promising opportunity to refine detection methodologies and identify new classes of environmental contaminants, thereby improving monitoring accuracy with significant implications for ecosystem health and sustainability. However, further experimental studies are required to optimize sampling protocols and translate these hypotheses into effective biomonitoring and conservation strategies.

## Data Availability

No new data were created or analyzed in this study.
